# Risk assessment model based on nucleotide metabolism-related genes highlights SLC27A2 as a potential therapeutic target in breast cancer

**DOI:** 10.1007/s00432-024-05754-x

**Published:** 2024-05-16

**Authors:** Bo Zhang, Yunjiao Zhang, Kexin Chang, Niuniu Hou, Pengyu Fan, Cheng Ji, Liuyin Liu, Zhe Wang, Ruolei Li, Yaping Wang, Jian Zhang, Rui Ling

**Affiliations:** 1 Department of Thyroid, Breast and Vascular Surgery, Xijing Hospital, Fourth Military Medical University, Xi’an, 710032 Shaanxi People’s Republic of China; 2 Department of General Surgery, Air Force 986(Th) Hospital, Fourth Military Medical University, Xi’an, 710032 Shaanxi People’s Republic of China; 3 Department of Thoracic Surgery, Tangdu Hospital, Fourth Military Medical University, Xi’an, 710038 Shaanxi People’s Republic of China; 4 The State Key Laboratory of Cancer Biology, Department of Biochemistry and Molecular Biology, Fourth Military Medical University, Xi’an, 710032 Shaanxi People’s Republic of China

**Keywords:** Breast cancer, Nucleotide metabolism, Cox regression, LASSO regression, Gene signature, SLC27A2

## Abstract

**Purpose:**

Breast cancer (BC) is the most prevalent malignant tumor worldwide among women, with the highest incidence rate. The mechanisms underlying nucleotide metabolism on biological functions in BC remain incompletely elucidated.

**Materials and Methods:**

We harnessed differentially expressed nucleotide metabolism-related genes from The Cancer Genome Atlas-BRCA, constructing a prognostic risk model through univariate Cox regression and LASSO regression analyses. A validation set and the GSE7390 dataset were used to validate the risk model. Clinical relevance, survival and prognosis, immune infiltration, functional enrichment, and drug sensitivity analyses were conducted.

**Results:**

Our findings identified four signature genes (DCTPP1, IFNG, SLC27A2, and MYH3) as nucleotide metabolism-related prognostic genes. Subsequently, patients were stratified into high- and low-risk groups, revealing the risk model's independence as a prognostic factor. Nomogram calibration underscored superior prediction accuracy. Gene Set Variation Analysis (GSVA) uncovered activated pathways in low-risk cohorts and mobilized pathways in high-risk cohorts. Distinctions in immune cells were noted between risk cohorts. Subsequent experiments validated that reducing SLC27A2 expression in BC cell lines or using the SLC27A2 inhibitor, Lipofermata, effectively inhibited tumor growth.

**Conclusions:**

We pinpointed four nucleotide metabolism-related prognostic genes, demonstrating promising accuracy as a risk prediction tool for patients with BC. SLC27A2 appears to be a potential therapeutic target for BC among these genes.

**Supplementary Information:**

The online version contains supplementary material available at 10.1007/s00432-024-05754-x.

## Introduction

Breast cancer (BC) stands as the predominant malignant tumor globally among women, with a high incidence and mortality rate. According to the recent report from the American Cancer Society, BC constitutes 31% of all new cancer diagnoses in women (Siegel et al. 2023). Despite advancements in treatment methods such as surgery, chemotherapy, targeted therapy, endocrine therapy, and immunotherapy, which have notably improved the overall 5-year survival rate to 90% for individuals diagnosed with localized early-stage BC, the survival rate for those with advanced BC remains below 30% (Waks and Winer 2019). Moreover, drug resistance poses a significant challenge to the efficacy of chemotherapy, radiotherapy, or endocrine therapy in certain BC cases. Given that BC development involves a multi-gene process, precise approaches are needed to overcome the limitations of conventional treatments. The application of genomic, transcriptomic, and proteomic research findings enables a more thorough assessment of the individualized biological characteristics of tumors, providing detailed information for evaluation and introducing new diagnostic methods and treatment targets for patients with BC.

Tumor onset is often accompanied by abnormal metabolic processes, and metabolic reprogramming plays an important role in tumor development, with nucleotide metabolism being a key player in these processes (Finley 2023). Nucleotide metabolism encompasses a variety of enzymes involved in the synthesis and decomposition of nucleotides, including purine and pyrimidine metabolism, which are fundamental building blocks of DNA/RNA synthesis. Purine and pyrimidine nucleotide synthetic pathways involve both de novo synthesis and salvage pathways of nucleosides/nucleobases (Hanahan and Weinberg 2011) (Robinson et al. 2020). Numerous studies have demonstrated that tumor proliferation, chemotherapy resistance, metastasis, and immune evasion are, in part, dependent on the overactivation of nucleotide metabolism reactions (Mullen and Singh 2023). Treatments targeting cancer cells’ metabolic abnormalities have become increasingly promising. For instance, 5-fluorouracil can inhibit thymidylate synthase, a key enzyme in the de novo synthesis of 2′-deoxythymidine-5′-monophosphate, representing a crucial aspect of cancer treatment (Peters et al. 2002). Beyond serving as fundamental substrates for nucleic acid synthesis, nucleotides also fuel various processes overly active in cancer cells. Consequently, targeting nucleotide metabolism holds significant promise as part of combination therapy strategies, an area that remains largely untapped.

Nucleoside analog drugs, such as Gemcitabine and Capecitabine, play a significant role in the clinical treatment of BC and various other cancer types. Concurrently, research indicates that the elevation in pyrimidine nucleotide levels after chemotherapy exposure reveals a metabolic target that can be used to improve the effectiveness of chemotherapy for triple-negative BC (TNBC). Consequently, pharmacologically inhibiting de novo pyrimidine production can sensitize TNBC cells to genotoxic chemotherapy drugs, thereby amplifying DNA damage (Brown et al. 2017). However, practical BC-specific nucleotide metabolism predictive models for assessing treatment outcomes and prognosis are currently lacking. Improving overall survival and disease-free survival is paramount in treating malignant tumors. Establishing personalized prognosis models with nucleotide metabolism-related genes (NMRGs) based on clinical samples for patients with BC is imperative for prognosis and cancer therapy.

In this study, we aimed to identify specific NMRGs to develop a prognostic model for predicting outcomes in patients with BC. We also sought to elucidate the landscape of biological pathways, immune infiltration, and predictions for immunotherapy and chemotherapy sensitivity. Consequently, we constructed a novel prognostic model using four signature genes (DCTPP1, IFNG, SLC27A2, and MYH3), forming a predictive signature with diagnostic value for patients with BC. Finally, through in vitro and in vivo experiments, we found that SLC27A2 could influence various biological functions of BC cells, including tumor nucleotide metabolism, lipid metabolism, and cell proliferation, establishing it as a valuable independent target for BC with significant research value.

## Materials and methods

### Data source

The BC dataset, comprising 1,080 BRCA samples and 113 normal samples, was extracted from TCGA database (https://portal.gdc.cancer.gov/). Additionally, the GSE7390 dataset (GPL96), consisting of 198 BRCA samples, was acquired from the Gene Expression Omnibus database (http://www.ncbi.nlm.nih.gov/geo/). A total of 2,334 NMRGs were obtained from the Comparative Toxicogenomics Database (http://ctdbase.org/).

### Selection and function enrichment of DEGs

In TCGA-BRCA cohort, the DESeq2 package (v 1.26.0) was utilized to identify DEGs between BRCA and normal cohorts, setting significance thresholds as |log2FC| > 1 and adj. *p* < 0.05 (Love et al. 2014). Volcano plots and heatmaps of DEGs expression were generated using ggplot2 (v 3.3.2) and Heatmap (v 4.1.0) (Gustavsson et al. 2022) (Gu and Hubschmann 2022). DEGs overlapping with NMRGs were further analyzed to identify differentially expressed-NMRGs (DE-NMRGs). Subsequently, DE-NMRGs were subjected to Gene Ontology (GO) and Kyoto Encyclopedia of Genes and Genomes (KEGG) enrichment study enrichment analysis using clusterProfiler (version 4.0.2) (adj. *p* < 0.05, count > 1). (Yu et al. 2012).

### Creation and verification of a risk model

The cohort of 1,080 patients with BRCA was randomly split into the training set (756 patients) and the validation set (324 patients) at a 7:3 ratio. Subsequently, univariate Cox analysis was conducted using expression data from DE-NMRGs in the training set (Ramsay et al. 2018). Then, it was checked whether the proportional hazards (PH) of the Cox model applied to each gene (*p* > 0.05). The Least Absolute Shrinkage and Selection Operator (LASSO) regression analysis, conducted through glmnet (v 4.0–2), was employed to identify signature genes (Friedman et al. 2010). The risk score was then determined using the equation:

$$\mathrm{risk score}={\sum }_{{\text{n}}=1}^{{\text{n}}}({\text{coefi}}*{\text{Xi}})$$

Here, Coef and X represent coefficients and gene expressions, respectively. Subsequently, patients in the training set, validation set, and GSE7390 dataset were stratified into high- and low-risk cohorts based on optimal thresholds for risk scores. Risk curves were plotted according to these scores. To assess prognosis differences, K–M survival analysis was conducted using survminer (v 0.4.6). To assess the predictive efficacy of the risk model, ROC curves for 1–5 years were plotted using the survivalROC package (v 1.0.3) (Heagerty et al. 2000). The risk model was further validated using the validation set and GSE7390 dataset. Finally, validation of signature gene expressions was performed in TCGA-BRCA dataset.

### Clinical correlation analysis of two risk cohorts

To identify the relationship between risk score expressions and clinical characteristics in BRCA, we categorized patients’ relevant clinical features into distinct subgroups based on factors such as age (> 55 and ≤ 55). Subsequently, we compared variations in patient risk scores across these clinical characteristic subgroups. Additionally, we performed a stratified survival analysis of the two risk cohorts across various clinical factors to gain further insights into the association between clinicopathological characteristics and survival outcomes. Finally, we assessed the expression of signature genes based on several clinical characteristics in these subgroups.

### Nomogram construction and independent prognostic analysis

Clinical characteristics (age, Tumor Mutational Burden (TMB), stage, etc.) and risk scores were included in univariate and multivariate Cox Regression analyses (*p* < 0.05) and proportional hazards assumption studies (*p* > 0.05) to further investigate the prognostic profile of clinicopathologic characteristics in relation to the risk model. After constructing a nomogram utilizing independent prognostic factors with the rms package (v 6.1.0), we evaluated the predictive power of the nomogram through ROC and calibration curves (Liu et al. 2021).

### Functional exploration in BC

Using hallmark gene sets, we conducted Gene Set Variation Analysis (GSVA) enrichment analysis on all genes in samples from the two risk cohorts. Limma (v 3.50.0) was employed to examine GSVA scores between the two risk cohorts in TCGA-BRCA dataset (Ritchie et al. 2015), with “*t*” chosen as the difference value. In the high-risk cohort, the pathway was considered activated at* t* > 1, while in the low-risk cohort, activation was at *t* < 1.

Furthermore, we conducted a differential assessment of the 13 carcinogenic pathways identified in the literature for the two risk cohorts in the training set (Xu et al. 2022). The relationship between signature genes and distinct carcinogenic pathways was further investigated. Additionally, Maftools (v 2.10.0) processed single nucleotide polymorphism mutation locus data from TCGA-BRCA patients to evaluate differences in mutations between the two risk cohorts (Mayakonda et al. 2018). Simultaneously, we computed TMB scores in the two risk cohorts using the same method. Subsequently, patients with BRCA were categorized into high and low TMB expression cohorts based on the median TMB score, and the combined two risk cohorts were used for survival analysis in TCGA-BRCA dataset.

### Immunoassay analysis and drug prediction

In the two risk cohorts, we calculated 28 immune cell scores using the single-sample gene set enrichment analysis algorithm of the GSVA package (v 3.0.3) (Hanzelmann et al. 2013). Comparisons of infiltrating immune cells in the two risk cohorts were conducted, and the connection between distinct immune cells and signature genes was determined. Stromal scores, immune scores, and ESTIMATE scores based on signature genes expression were generated using the ESTIMATE program, and differences between the two risk cohorts were assessed (Yoshihara et al. 2013). We assessed the expression of immune checkpoints in the two risk cohorts based on various types described in the literature and determined the link between the top 10 differentially expressed immune checkpoints, signature genes, and risk score (Li et al. 2021).

To explore the response to immune checkpoint inhibitors in both risk cohorts, we calculated the Tumor Immune Dysfunction and Exclusion (TIDE) score (http://tide.dfci.harvard.edu). We obtained data on the corresponding immunophenoscore (IPS) of patients with BRCA from The Cancer Imaging Archive (TCIA) database for intergroup difference analysis (https://www.cancerimagingarchive.net).

The half maximal inhibitory concentration (IC50) values for 56 drugs in BRCA samples were derived using pRRophetic (v 0.5) (Geeleher et al. 2014). The association between risk score and IC50, as well as signature genes and IC50 (top 10), were evaluated (|R|> 0.4, *p* < 0.05).

### Cell culture

The MCF7, Hs 578T, AU565, MDA-MB-361, and HEK293T cell lines were cultured in DMEM (11965092, Gibco, USA), supplemented with 10% FBS (A5669701, Gibco, USA), and 1% streptomycin/penicillin (C0222, Beyotime, China). The HCC1937, BT-474, BT-549, and 4T1 cell lines were cultured in RPMI 1640 medium (31800022, Gibco, USA), supplemented with 10% FBS (A5669701, Gibco, USA), and 1% streptomycin/penicillin (C0222, Beyotime, China).MCF 10A was cultured in special culture medium (CM-0525, Pricella, China). The Shanghai Cell Bank of the Chinese Academy of Sciences provided all cell lines mentioned above. Cells were in logarithmic growth phase during experiments and the cell passage numbers between repeated experiments did not exceed 5 generations. We cultivated the cells at 37 °C with 5% CO_2_.

### In vivo experiments

To elucidate the role of SLC27A2 in vivo, 5-week-old female BALB/c mice from the Experimental Animal Center of the Fourth Military Medical University were employed. 4T1 cells, resuspended in RPMI 1640 medium, were subcutaneously injected into the mammary fat pad of each mouse at a dose of 2 × 10^6^ cells to establish the BC model (*n* = 6 mice per group). Tumor volume was assessed using the formula V = 1/2 × L × W^2^ (V: volume, L: length, W: width). Once tumors reached a size of approximately 100 mm^3^, mice were administered intraperitoneal injections of Lipofermata (Adeshakin et al. 2021) at a dose of 2.5 mg/kg every other day for 2 weeks. Tumor volumes and body weights were measured every 3 days in each group until the mice were euthanized, at which point the tumors were excised. All experimental procedures received approval from the Animal Care and Use Committee of the Fourth Military Medical University.

### Clinical samples

BC and paracancerous tissue microarray samples were obtained from Sinochem Guanghua (Xi'an) Intelligent Biotechnology Co., Ltd. After fixing in 4% paraformaldehyde for 24 h, the specimens were embedded in paraffin and analyzed by immunohistochemical (IHC). The tissue microarray was incubated with SLC27A2 (1:200, sc-393906, Santa Cruz, USA) primary antibody at an appropriate concentration. Images were collected using Caseviewer 2.2.1 software.

### shRNA and lentiviral production

The human and mouse SLC27A2 lentivirus shRNA and control shRNA were procured from Beijing Qingke Biotechnology Co., Ltd. The shRNA sequence was constructed as pLKO.1-PURO and co-transfected into HEK293T cells with pMD2.G and psPAX2 plasmids to produce lentiviral particles. Culture supernatants containing lentivirus particles were collected at 48 and 72 h post-transfection. MCF7, HCC1937, and 4T1 cells were infected with lentivirus approximately 24 h after the medium was changed, and then screened with puromycin to isolate the lentivirus stable strains. The sequences of shRNA used were as follows: h-shSLC27A2 #1: CCATACTTCTTCCAGGACATA; h-shSLC27A2 #2: GCTGATTACCTACCTAGTTAT; m-shSLC27A2 #1: GCGTGCCTCAACTACAACATT; m-shSLC27A2 #2: GCAGAACTTCTAACACAAATG.

### Western blot

Cells were lysed using RIPA buffer (P0013B, Beyotime, China) containing protease and phosphatase inhibitors (B14001, Selleck, USA), and protein levels were quantified using the BCA protein assay kit (P0011, Beyotime, China). Equivalent amounts of total protein per lane were loaded, separated by SDS-PAGE gel, transferred to PVDF membranes (Millipore, USA), and blocked in TBST buffer with 5% (w/v) skim milk at room temperature. PVDF membranes were incubated overnight at 4 °C with primary antibodies. The secondary antibodies conjugated with peroxidase were detected by chemiluminescence (Tanon 5200 image system), and β-Actin and GAPDH expression were used as controls. The antibodies used are listed in Supplementary Table S1.

### qPCR analysis

RNA isolation and purification were performed after two washes with PBS. Total RNA was extracted with RNAiso Plus (9109, TaKaRa, Japan), and then used for cDNA synthesis (15,662-A, Yeasen, China). The qPCR analysis was performed using SYBR Green PCR Master Mix (11201ES50, Yeasen, China) and an ABI QuantStudio 5 Real-Time PCR System Instrument. Data were analyzed using the 2^–ΔΔCt^ method and calculated using ACTB as a normalized control. The primer sequences (Occo Dingsheng Biotechnology, Beijing, China) are listed in Supplementary Table S2.

### CCK-8 assay

We plated 4 × 10^3^ cells per well in 96-well plates: MCF7, HCC1937 shNC, shSLC27A2-1, and shSLC27A2-2. After 24 h, 48 h, 72 h, and 96 h, cells were treated with 10% CCK-8 solution (40203ES60, Yeasen, China) and then incubated at 37 °C for 1 h. Subsequently, the absorbance was measured at 450 nm using a microplate reader (BioTek, USA) to assess cell viability. To evaluate the effects of Lipofermata on the MCF 10A, BT-474, MCF7, HCC1937, and MDA-MB-361 cell lines, the CCK-8 kit was utilized. MCF 10A, BT-474, MCF7, HCC1937, and MDA-MB-361 cell lines were plated in 96-well plates at a concentration of 4 × 10^3^ cells. Subsequently, the cells were exposed to Lipofermata at a maximum concentration of 4 μM for 48 h. Then, the CCK-8 procedure was the same as above. Finally, the IC50 value was calculated using GraphPad Prism 9.5 software.

### Immunofluorescence and lipid uptake microscopy

Cells were cultured on 15 mm glass-bottom dishes. Cells were starved for 6 h in serum-free medium to assess lipid uptake under intervention or inhibition of SLC27A2. The control group, the shSLC27A2 group, and the drug-treated group were then cultured in DMEM with 2% FBS and 1:100 Lipid Mixture 1 (L0288, Sigma-Aldrich, USA). Either DMSO or Lipofermata was added for 24 h. Prior to fixation with 4% (v/v) paraformaldehyde, the cells were incubated with 1 μM BODIPY 558/568 C-12 (D3835, Invitrogen, USA) for 2 h. The cells were permeabilized with 0.1–0.2% (v/v) TritonX-100 (ST795, Beyotime, China) in PBS for 10 min, followed by washing and blocking with 5% (w/v) bovine serum albumin (BSA) for an additional hour. The fixed cells were incubated with the primary antibody SLC27A2 (1:500, sc-393906, Santa Cruz, USA) overnight at 4 °C. The next day, we washed the cells and incubated them with FITC-labeled secondary antibodies (1:100, Boster, China) for 1 h at ambient temperature. DAPI (C1002, Beyotime, China) was used to stain the nuclei. Subsequently, the images were captured using a fluorescence confocal microscope from Olympus, Japan.

### IHC staining

Paraffin-embedded tissue sections and the tissue microarray slide underwent IHC staining. For antigen repair, tissue sections were placed in a repair box containing citric acid antigen repair buffer in a microwave oven, and blocked with 3% hydrogen peroxide for 20 min. Blocking was performed with 5% BSA for 15 min at room temperature. After removing the blocking solution, the slides were incubated with the SLC27A2 primary antibody (1:200, sc-393906, Santa Cruz, USA) at 4 °C overnight. The next day, tissues were covered with secondary antibodies and incubated for 1 h at room temperature. Then, 3,3′-tetrachloride diaminobenzidine was used for color development. Images were obtained using a microscope. In IHC, the percentage of staining tumor cells was calculated by two independent pathologists (0%: 0; < 25%: 1; 25–50%:2; 50–75%: 3; > 75%: 4), and the degree of staining intensity was also recorded (no staining: 0; weak staining: 1; moderate staining: 2; strong staining: 3). IHC staining score = the percentage of stained tumor cells × staining intensity.

### Cell cycle and apoptosis assay

5 × 10^5^ cell were seeded per well in six-well tissue culture plates for the cell cycle experiments. After the cells adhered to the substrate, they were either subjected to starvation treatment or simultaneously treated with a lipid mixture. Cells were collected and fixed with precooled 70% (v/v) ethanol overnight, followed by incubation with RNase and propidium iodide (PI) to observe cell cycle progression. To determine the percentage of cells in G0/G1, S, and G2/M phases, flow cytometry was used (BD Biosciences, USA). The Annexin V-FITC/PI apoptosis detection kit (640914, Biolengend, USA) was used to evaluate apoptotic cells. At room temperature for 15 min, cells were stained with 5 μL of Annexin V-FITC solution and 10 μL of PI solution, then detected by flow cytometry (BD Biosciences, USA).

### EdU cell proliferation assay

Proliferation of cells was determined using the EdU staining test (C0075S, Beyotime, China). MCF7 and HCC1937 cells were seeded in six-well plates and exposed to 10 μM EdU for 2 h, then fixed with 4% paraformaldehyde for 15 min and permeabilized with PBS containing 0.3% Triton X-100 for 15 min at ambient temperature. The following staining steps were carried out following the manufacturer's guidelines and finally detected by a fluorescence microscope (Nikon, Japan).

### Statistical analysis

Bioinformatics analyses were executed in the R language. All the above experiments were performed in triplicate. To analyze the significant differences between groups, either Student's *t*-test or one-way ANOVA was utilized. The experimental data were processed and analyzed using GraphPad Prism 9.5. Differences were considered statistically significant when the *p*-value was less than 0.05.

## Results

### Identification and functional exploration of DE-NMRGs in BC

**Fig. 1:**
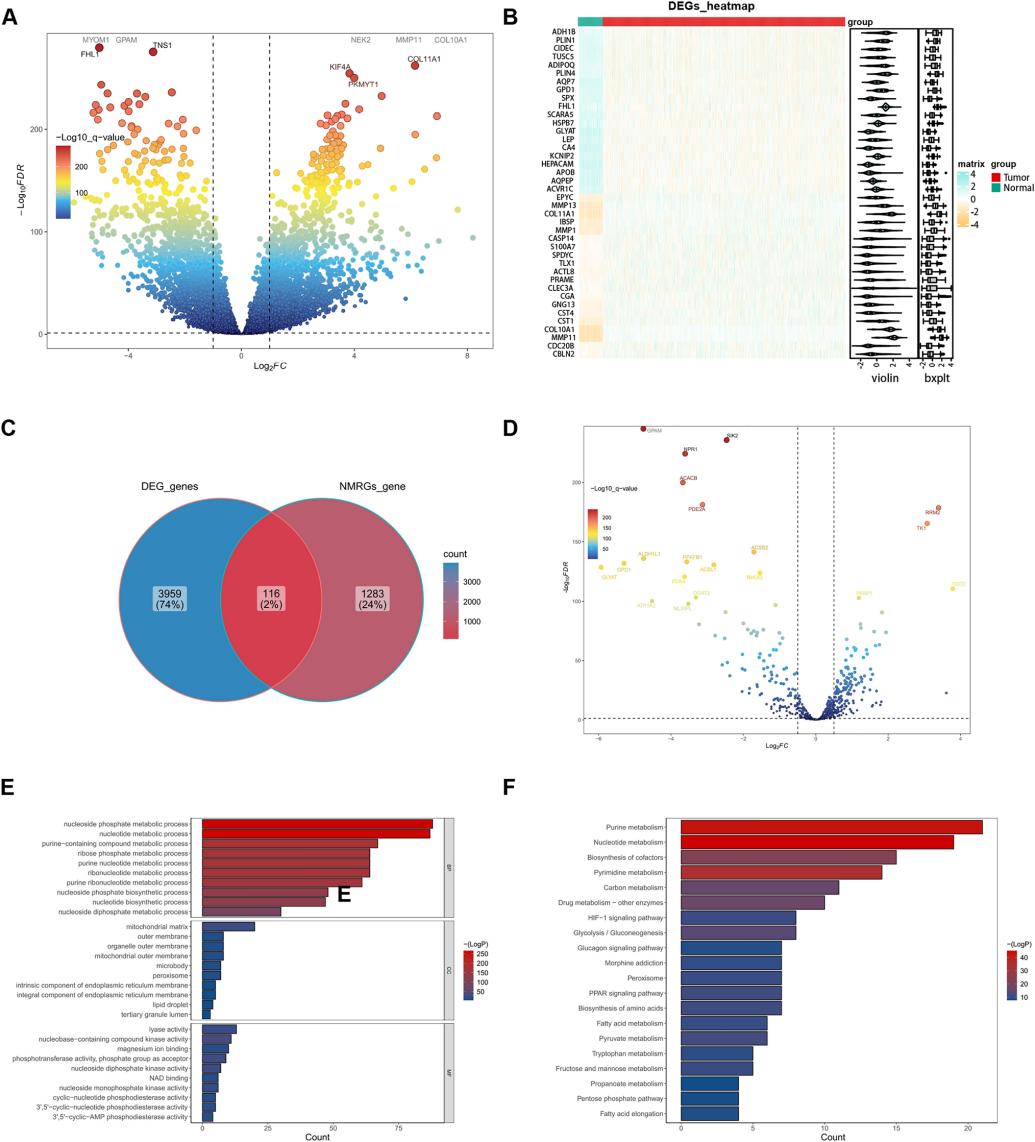
Identification and functional exploration of DE-NMRGs in BC. **A** Identification of differentially expressed genes in BC. Volcano plot illustrates DEGs in normal samples versus tumor samples from the TCGA dataset. **B** Heat map representation of the differential expression of genes, highlighting expression density. **C** Venn diagram displaying the overlapping genes between DEGs in BC and NMRGs. **D** DE-NMRGs showing in volcano plot. **E** The bar graph presenting the GO enrichment analysis result of DE-NMRGs. **F** The bar graph displaying the KEGG enrichment analysis result of DE-NMRGs

To obtain DE-NMRGs in BC, we conducted differential expression analysis between BC and normal cohorts in The Cancer Genome Atlas (TCGA)-BRCA dataset based on gene selection strategies and bioinformatics methods (Zhang et al. 2018). In total, 4075 differentially expressed genes (DEGs) (2347 enhanced and 1728 diminished DEGs) were identified in TCGA-BRCA dataset (Fig. 1a). And the top 20 of differentially expressed genes was shown in the heatmap (Fig. 1b). Subsequently, DEGs between BRCA and normal cohorts were overlapped with NMRGs to screen 116 DE-NMRGs in BRCA (Fig. 1c, d).

Research from the GO biological process revealed a significant enrichment of DE-NMRGs in the “nucleoside phosphate metabolic process”, “nucleotide metabolic process” and “purine-containing compound metabolic process”. According to GO cell composition, DE-NMRGs were predominantly associated with the “mitochondrial matrix”, “outer membrane” and “organelle outer membrane” In terms of molecular function, DE-NMRGs were primarily linked to “lyase activity”, “nucleobase-containing compound kinase activity” and “magnesium ion binding” as identified by GO molecular function (Fig. 1e). Finally, DE-NMRGs were mostly associated with “purine metabolism”, “nucleotide metabolism” and “biosynthesis of cofactors” as presented in the KEGG (Fig. 1f).

### Construction and assessment of the DE-NMRGs prognostic signature

**Fig. 2:**
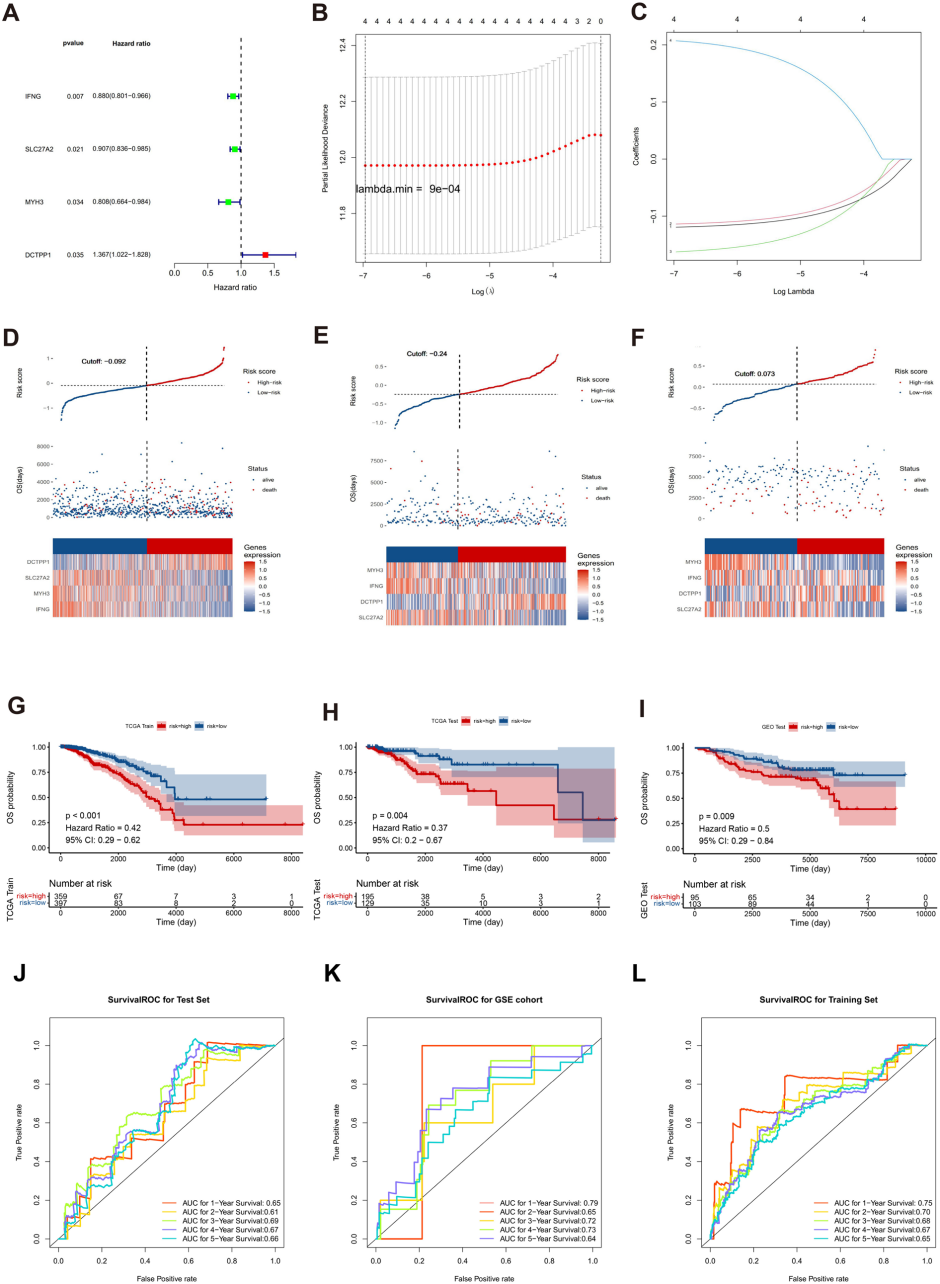
Development and validation of the DE-NMRGs signature. **A** Forest plot illustrating the four signature genes (DCTPP1, IFNG, SLC27A2, MYH3) identified through univariate Cox regression analysis. **B** Selection of four candidate genes via LASSO regression. **C** The trace of each individual gene. **D**–**F** Representation of risk score, survival status, and expression patterns of the four genes in the training set, validation set, and GSE7390 dataset, respectively. **G**–**I** Kaplan–Meier (K-M) survival analysis illustrating the overall survival (OS) of patients in high- and low-risk groups in the training set, validation set, and GSE7390 dataset. **J**–**L** Receiver operating characteristic (ROC) curves for predicting 1-, 2-, 3-, 4-, and 5-year survival based on risk scores in the training set, validation set, and GSE7390 dataset

The univariate Cox regression analysis yielded four signature genes (DCTPP1 as a risk factor and IFNG, SLC27A2, and MYH3 as protective factors) of BC in the training set (Fig. 2a). The Schoenfeld test revealed that all four genes were validated (Fig. S1a–d). In the LASSO regression analysis, with λ = 0.00009 and employing tenfold cross-validation, the LASSO regression model identified DCTPP1, IFNG, SLC27A2, and MYH3 as important predictors with non-zero coefficients (Fig. 2b, c). Risk scores were computed by coefficients and signature gene expression in BC samples. In the training set, validation set, and GSE7390 dataset, the allocation of survival status and risk scores were assessed after classifying individuals into high- and low-risk cohorts using optimal thresholds for the risk score (Fig. 2d–f). The Kaplan–Meier (K–M) curves for the training set, validation set, and GSE7390 dataset demonstrated a marked difference in survival outcomes between the two risk cohorts (Fig. 2g–i). The area under the receiver operating characteristic curve of the risk model for 1–5 years in the three datasets were all greater than 0.65, revealing that this model possessed great predictive efficacy (Fig. 2j–l).

### Assessment of independent prognostic factors in BC

**Fig. 3:**
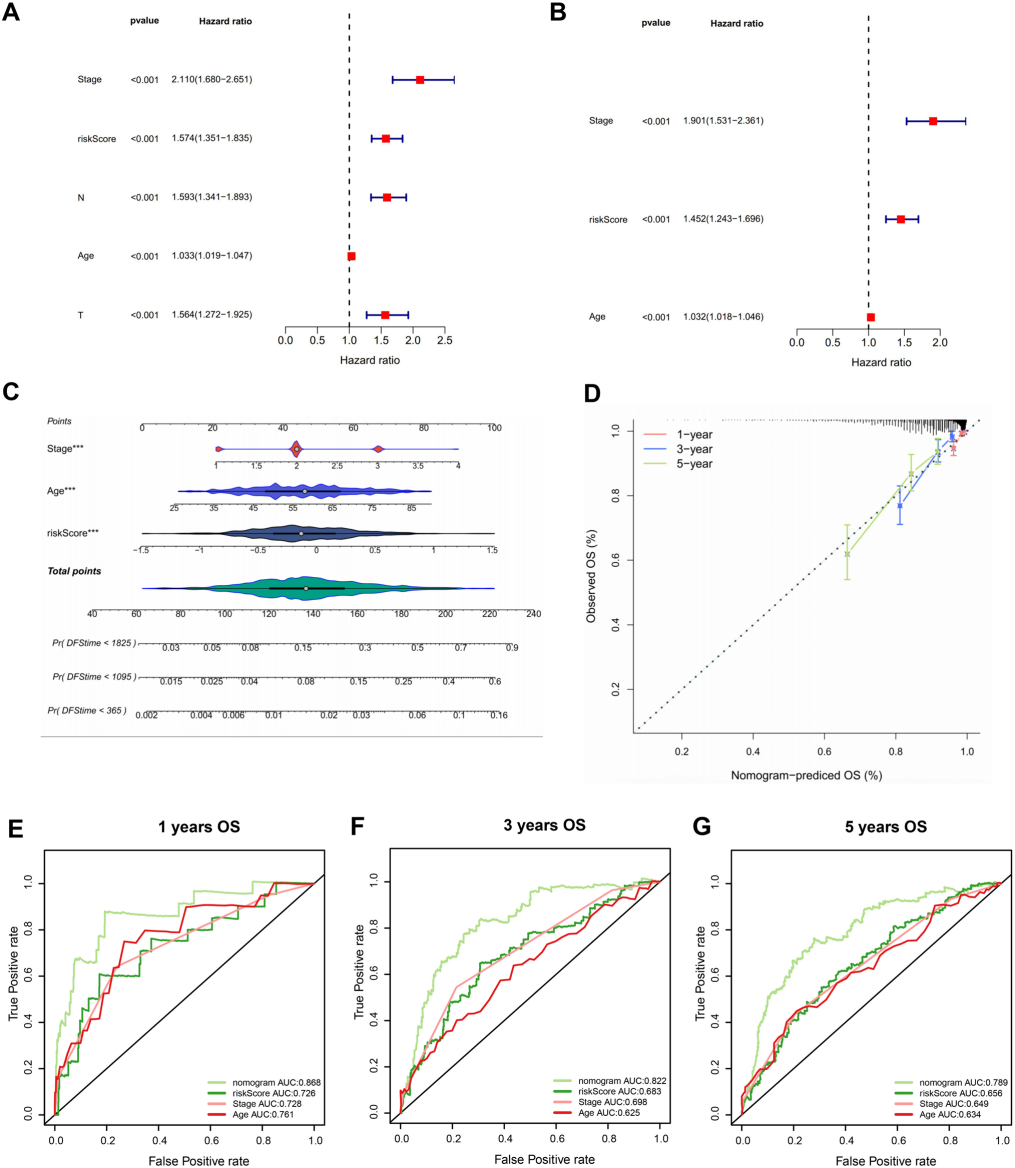
Evaluation of independent prognostic factors in BC. Univariate (**A**) and multivariate (**B**) Cox regression analyses of the signature genes for overall survival (OS) in breast cancer. **C** Prognostic nomogram integrating gene risk score and clinical prognostic factors. **D** Calibration plots demostrate the superb conformity of the 1-year, 3-year, and 5-year predicted lines with the nomogram predictions. The signature demonstrated superior predictive performance compared to other clinical parameters, with area under the receiver operating characteristic (ROC) curve (AUC) values of 0.868, 0.822, and 0.789 for 1-year (**E**), 3-year (**F**), and 5-year (**G**) overall survival (OS), respectively

To assess the prognostic capability of the risk signature, we incorporated clinical pathological factors into the risk model for univariate and multivariate Cox regression analysis. The results demonstrated that the signature exhibited independent prognostic significance in both univariate and multivariate Cox regression analyses (Fig. 3a, b). As illustrated in Fig. 3c, prognostic models developed via independent prognostic factors were visualized by nomogram. Calibration curve results suggested that the slope was close to 1 for 1, 3, and 5 years, indicating superior prediction accuracy of the model (Fig. 3d). Moreover, the enhanced prediction ability of the prognostic model was supported by the ROC, with AUC values greater than 0.6 (Fig. 3e–g).

### Function and mutation exploration in BC

**Fig. 4:**
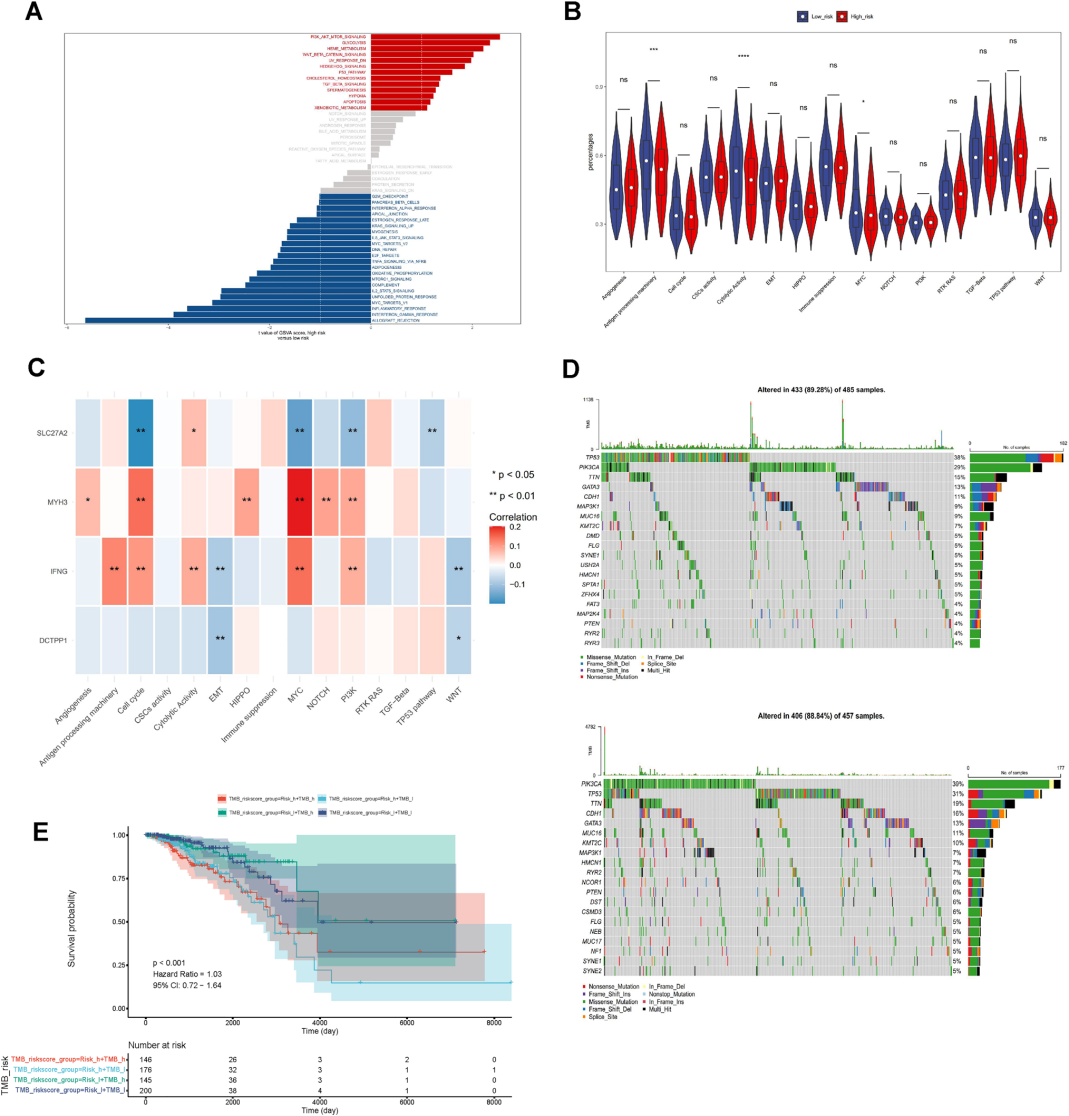
Exploration of function and mutations in BC. **A** Functional enrichment analysis conducted using GSVA. **B** Violin plot presenting the distribution of carcinogenic pathways in the low- and high-risk sets. **C** The expression correlation between the carcinogenic pathways and the 4 prognostic genes. **D** Waterfall plot depicting the Tumor Mutational Burden (TMB) of 20 genes in the high-risk set and low-risk set. **E** Survival analysis of overall survival (OS) among the Risk_high + TMB_high, Risk_high + TMB_low, Risk_low + TMB_high, and Risk_low + TMB_low cohorts in BRCA patients (*p < 0.05, **p < 0.01, ***p < 0.001)

Based on GSVA enrichment results, 22 pathways, including “inflammatory response”,  “interferon gamma response” and “allograft rejection” were significantly activated in the low-risk cohort. In contrast, 13 pathways, including “PI3K AKT mTOR signaling”,  “glycolysis,” and “heme metabolism” were considerably mobilized in the high-risk cohort (Fig. 4a). Carcinogenic pathways investigation in TCGA-BRCA revealed substantial differences between the two risk cohorts in “antigen processing machinery” , “cytolytic activity” and the “MYC pathway” (Fig. 4b). Spearman correlation analysis demonstrated that the IFNG was favorably connected with the three significantly distinct carcinogenic pathways, and the MYC pathway was positively and negatively associated with MYH3 and SLC27A2, respectively (Fig. 4c).

The TCGA-BRCA mutation data were compiled to compare the gene mutations between the two risk groups. In Fig. 4d, 89.28% of the high-risk cohort and 88.84% of the population in the low-risk cohort possessed mutations. Missense mutations predominated among mutation types in both cohorts, and the tumor mutational burden (TMB) index was generally greater in the high-risk cohort than in the low-risk cohort. Patients with BC were split into high and low TMB expression cohorts according to the median TMB score, and survival analysis combined with the two risk cohorts indicated survival differences among the four cohorts (Fig. 4e). In either the high or low TMB group, the high-risk group had worse prognosis than the low-risk group (Fig. S2a, b).

### Investigation of the immune microenvironment in BC

**Fig. 5:**
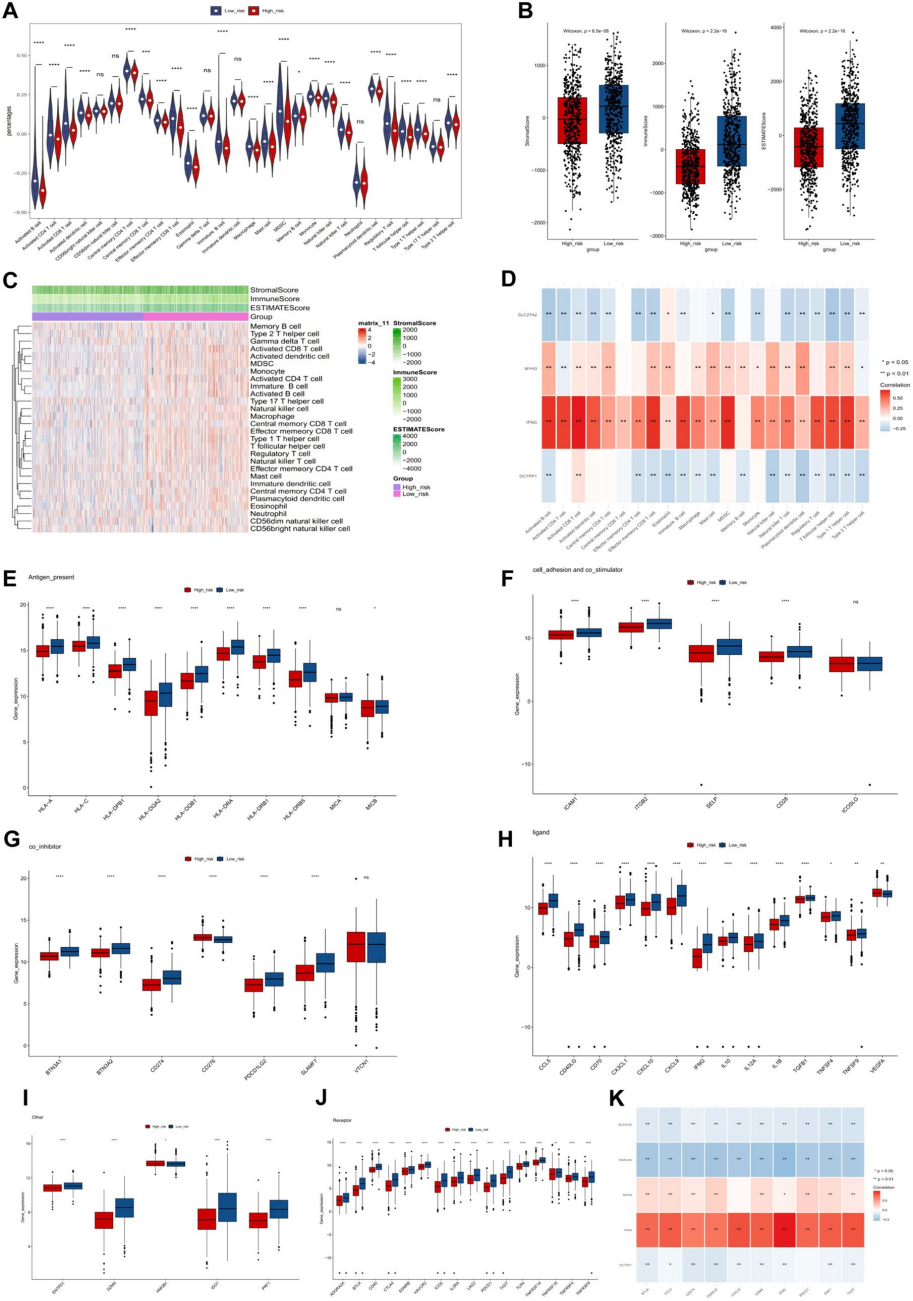
Investigation of the immune microenvironment in BC. **A** Violin plot displaying the distribution of 22 types of tumor-infiltrating immune cells between two risk cohorts in the TCGA-BRCA dataset. **B** Analyzing stromal scores, immune scores, and overall ESTIMATE scores in low- and high-risk patients utilizing the ESTIMATE algorithm. **C** The heat map shows the relative proportions of 28 immune cell subsets in low-risk and high-risk groups, as well as different stromal scores, immune scores, and ESTIMATE scores. **D** The correlation between the immune cells and the signature genes. **E**–**J** Assessment of differences in 57 immune checkpoints between two risk cohorts. **K** Heatmap displays the top 10 immune checkpoints closely correlated with IFNG, SLC27A2, MYH3 and risk scores in BC (*p < 0.05, **p < 0.01, ***p < 0.001, ****p < 0.0001)

Substantial variances were noted in 22 immune cells between the two risk cohorts of TCGA-BRCA dataset, including central memory CD4^+^ T cells, activated CD8^+^ T cells, memory effector CD4^+^ T cells, and eosinophils (Fig. 5a). The stromal scores and ESTIMATE scores were notably lower in the high-risk cohort compared to the low-risk cohort (Fig. 5b, c). Additionally, the relevance between differential immune cells and signature genes revealed that IFNG and MYH3 were significantly positively related to the majority of immune cells, while DCTPP1 and SLC27A2 exhibited negative correlation (Fig. 5d). Substantial differences between the two risk cohorts were observed in 57 immune checkpoints (Fig. 5e, j). The top 10 differential immune checkpoints were screened for correlation with signature genes and risk score calculation. The findings demonstrated that the top 10 immune checkpoints were positively linked to MYH3 and IFNG and negatively linked to DCTPP1, SLC27A2, and risk scores, respectively (Fig. 5k).

### Responsiveness to immunotherapy and drug forecasting in BC

**Fig. 6:**
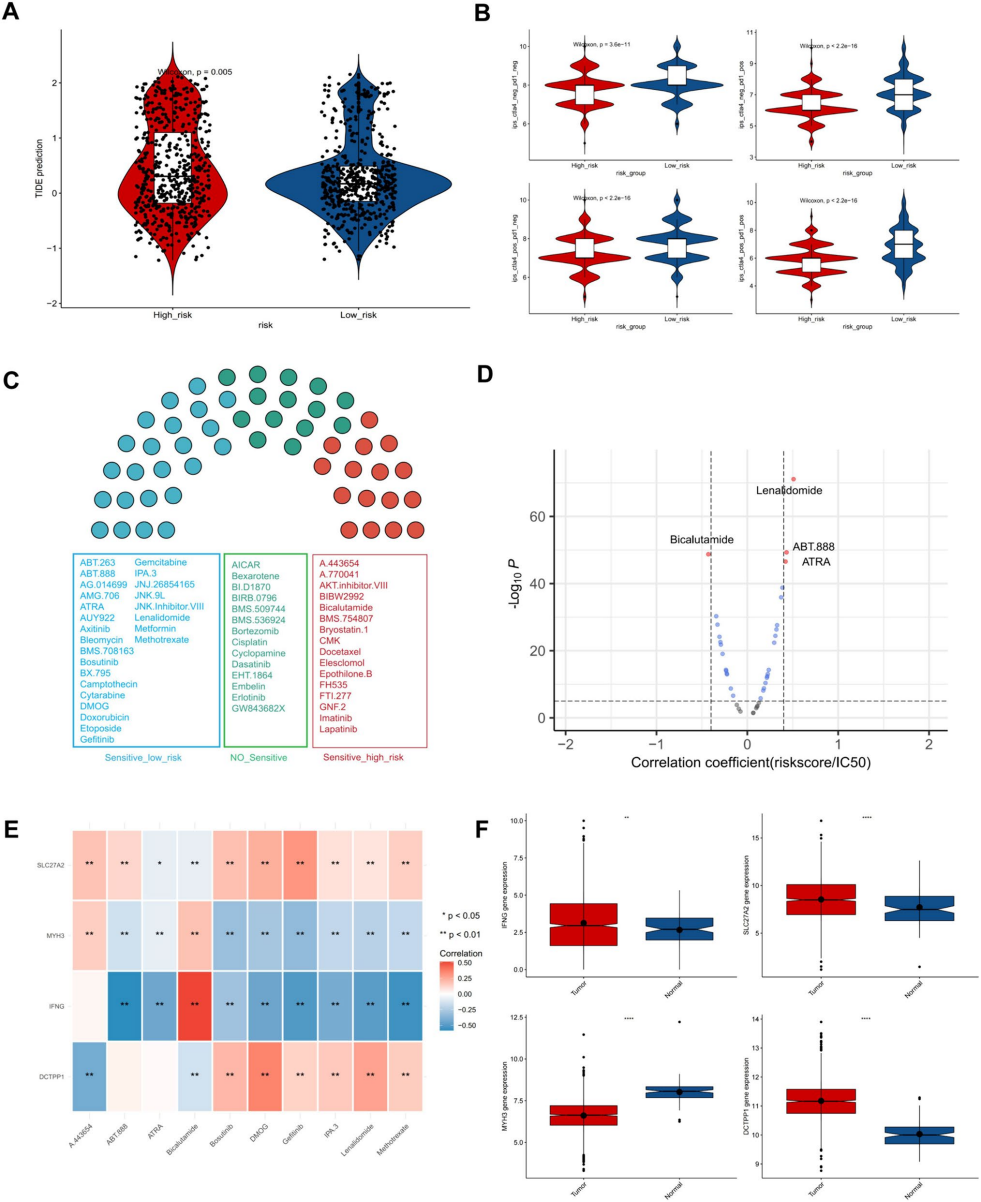
Evaluation of responsiveness to immunotherapy and drug forecasting in BC. **A** Assessment of TIDE score in high-risk and low-risk groups with significant statistical differences between the two subgroups determined using the Wilcoxon test. **B** Four subtypes of IPS values (ips_ctla4_neg_pd1_neg, ips_ctla4_neg_pd1_pos, ips_ctla4_pos_pd1_neg, and ips_ctla4_pos_pd1_pos) in high-risk and low-risk groups. **C** Variations in the responsiveness of chemotherapeutic agents between the two risk cohorts. **D** Correlation coefficients of Lenalidomide, ABT.888, ATRA and Bicalutamide with risk scores. **E** Heatmap illustrating the correlation between chemotherapeutic agents and SLC27A2, MYH3, IFNG, and DCTPP1. **F** The mRNA expression levels of IFNG, SLC27A2, MYH3 and DCTPP1 in TCGA-BRCA

The high-risk cohort demonstrated markedly elevated TIDE scores in contrast to the low-risk cohort (Fig. 6a). IPS outcomes in the two risk cohorts demonstrated substantial differences in ips_ctla4_neg_pd1_neg, ips_ctla4_neg_pd1_pos, ips_ctla4_pos_pd1_neg, and ips_ctla4_pos_pd1_pos groups (Fig. 6b). A notable difference in the sensitivity of 41 chemotherapeutic agents between the two risk cohorts was observed, with 16 drugs showing sensitivity in the high-risk cohort (such as A.443654, FH535, and Bicalutamide) and 25 in the low-risk cohort (such as Lenalidomide, Methotrexate, and ABT.888) (Fig. 6c). Among them, the correlation coefficients of Lenalidomide, ABT.888, and ATRA with risk scores were greater than 0.4, and Bicalutamide was − 0.42 (Fig. 6d). Lenalidomide, Methotrexate, ABT.888, ATRA, DMOG, IPA.3, and Gefitinib were significantly inversely associated with IFNG and MYH3 and strongly positively associated with DCTPP1 (Fig. 6e). In TCGA-BRCA database, DCTPP1, SLC27A2, and IFNG were highly expressed in cancer tissues, while MYH3 was expressed at a low level in cancer tissues (Fig. 6f).

### Validation of four NMRGs in BC

**Fig. 7:**
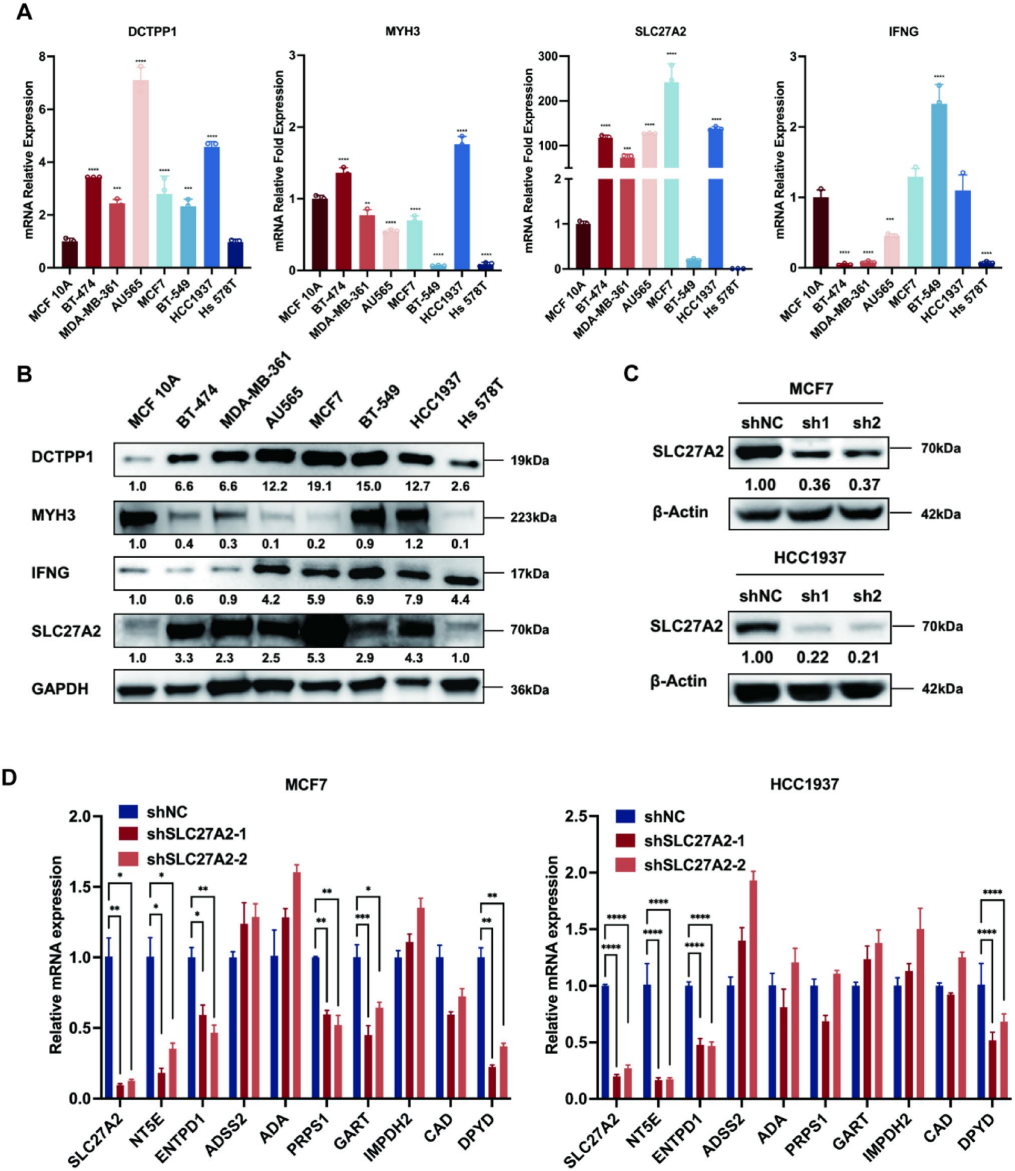
Validation of four NMRGs in BC. The mRNA (**A**) and protein (**B**) expression levels of SLC27A2 in mammary epithelial cell line (MCF 10A) and breast cancer cell lines (BT-474, MDA-MB-361, AU565, MCF7, BT-549, HCC1937, and Hs 578T) (**C**) SLC27A2 protein expression in SLC27A2 knockdown MCF7 and HCC1937 cell lines. **D** qRT-PCR analysis demostrates the mRNA levels of nucleotide metabolism-related genes in the two SLC27A2 knockdown cell lines. Data are presented as Mean ± SD (n = 3); statistical significance was determined using one-way ANOVA with Dunnett’s multiple comparisons test; *p < 0.05, **p < 0.01, ***p < 0.001, ****p < 0.0001

To explore the expression levels of MYH3, IFNG, DCTPP1, and SLC27A2, quantitative reverse transcription PCR (qRT-PCR) and Western blot assays were used to evaluate mRNA and protein expression. We compared the mammary epithelial cell line (MCF 10A) with BC cell lines (BT-474, MDA-MB-361, AU565, MCF-7, BT-549, HCC1937, and Hs 578T). In qPCR experiments, DCTPP1 mRNA expression was upregulated in BC cell lines except for Hs 578T, while MYH3 was downregulated in MDA-MB-361, AU565, MCF-7, BT-549, and Hs 578T. SLC27A2 expression was upregulated in BT-474, MDA-MB-361, AU565, MCF-7, and HCC1937 cell lines, while IFNG was upregulated in MCF7, BT-549, and HCC1937 (Fig. 7a). The protein levels of DCTPP1, IFNG, and SLC27A2 were markedly higher in almost all BC cell lines than in the normal mammary epithelial cell line, while MYH3 was lower expressed in BC cell lines, which was nearly consistent with the expressive analysis result from TCGA-BRCA database (Fig. 7b). Since SLC27A2 is significantly upregulated at both the RNA and protein levels in BC cell lines, and its biological functions and roles in BC have not been reported, we chose SLC27A2 for further investigation. Notably, MCF7 and HCC1937 cells exhibited relatively higher expression of SLC27A2. To investigate the possible biological function of SLC27A2 in BC, we used MCF7 and HCC1937 cell lines stably transduced with SLC27A2-shRNA and confirmed the downregulated expression level using Western blot (Fig. 7c). The expression of genes related to nucleotide metabolism, including NT5E, ENTPD1, and DPYD, was found to be reduced upon silencing SLC27A2 expression (Fig. 7d).

### Knockdown of SLC27A2 inhibits BC cell proliferation and cell cycle

**Fig. 8:**
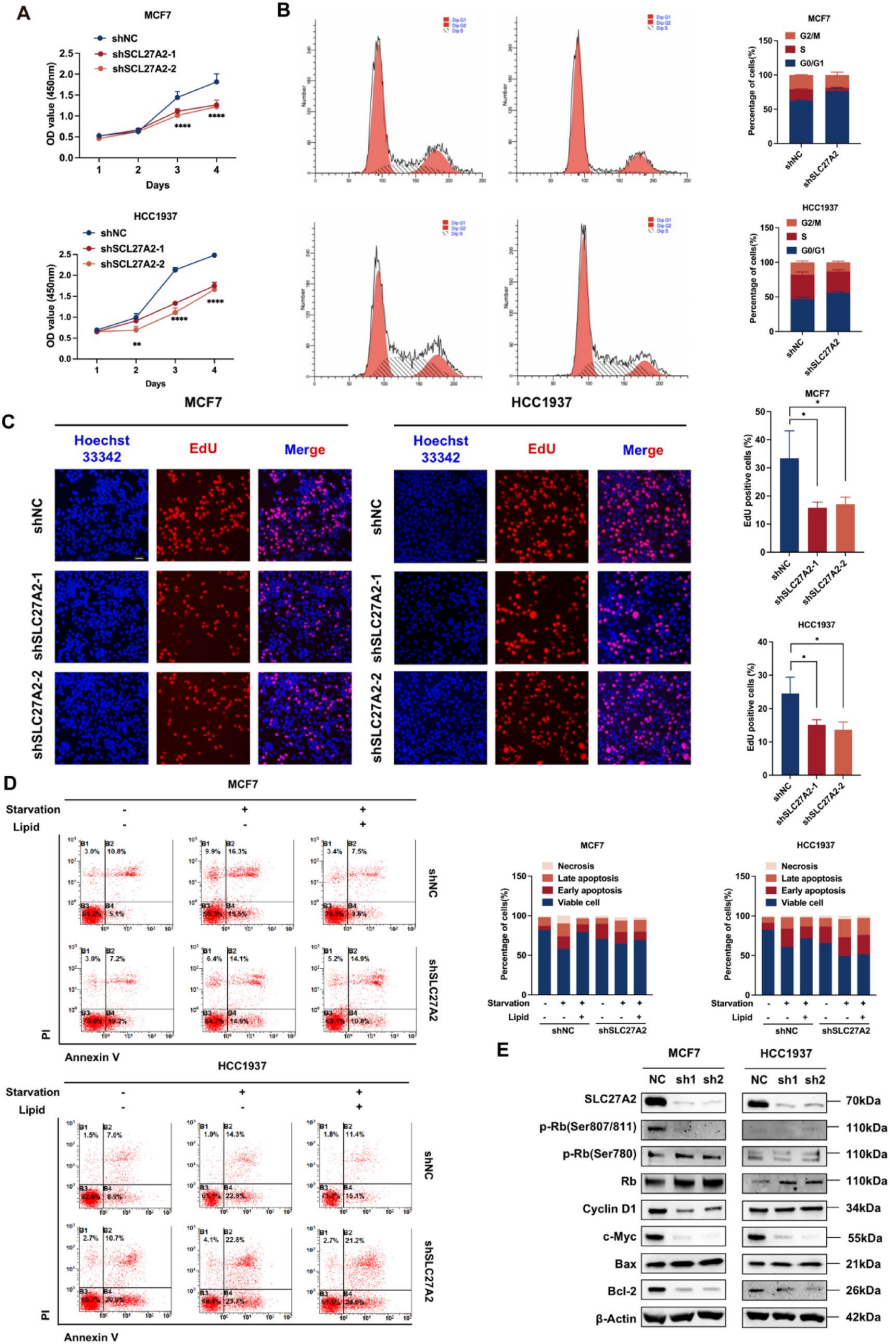
Knockdown of SLC27A2 inhibits BC cell proliferation and cell cycle. **A** CCK-8 assay shows cell growth curves of MCF-7 and HCC1937 and their SLC27A2 knockdown cell lines. Mean ± SD (n = 3); two-way ANOVA with Dunnett’s multiple comparisons test; **p < 0.01, ****p < 0.0001. **B** Flow cytometry cell cycle assay indicated changes in the different phases of the cell cycle in MCF7 and HCC1937 cells, along with their SLC27A2 knockdown counterparts. **C** Evaluation of cell proliferation in MCF7 and HCC1937 cells, and their SLC27A2 knockdown cell lines, using EdU (red) and Hoechst (blue) staining. The percentage of EdU-positive cells was calculated as EdU-positive cells/Hoechst-positive cells. Data are presented as Mean ± SD (n = 3); statistical significance is determined using one-way ANOVA with Dunnett’s multiple comparisons test; *p < 0.05. **D** Flow cytometry analysis of cell apoptosis in shNC and shSLC27A2 cells cultured in complete media, delipidized media, and delipidized media supplemented with fatty acids. A representative experiment is shown from n = 3 independent experiments. **E** Cell cycle and apoptotic biomarkers in MCF7 and HCC1937 cells with SLC27A2 knockdown

The CCK-8 assay revealed that silencing SLC27A2 expression led to a significant reduction in the proliferation of both MCF7 and HCC1937 cells (Fig. 8a). We speculated that the growth inhibition of cells might be related to the cell cycle, so we conducted the study to explore the impact of SLC27A2 on the cell cycle. Flow cytometry was performed following PI staining, which indicated that knocking down SLC27A2 could decrease the proportion of cells in S phase (Fig. 8b). Moreover, knockdown of SLC27A2 decreased the percentage of EdU-positive cells in the EdU assay (Fig. 8c). Knocking down SLC27A2 increased the proportion of apoptotic cells in BC, and providing long-chain fatty acids (FAs) under cellular starvation conditions could rescue starvation-induced cell death but could not rescue the death of SLC27A2 knocked-down cell lines, indicating that knocking down SLC27A2 may lead to disturbances in cellular lipid uptake and transport (Fig. 8d). Through Western blot analysis, we found that knocking down SLC27A2 downregulated the expression of cyclin D1 and c-Myc, thereby affecting the cell cycle. Additionally, Bcl-2/Bax ratio was down-regulated, consequently increasing cell apoptosis (Fig. 8e).

### FATP2 blockade inhibits BC growth and lipid uptake

**Fig. 9:**
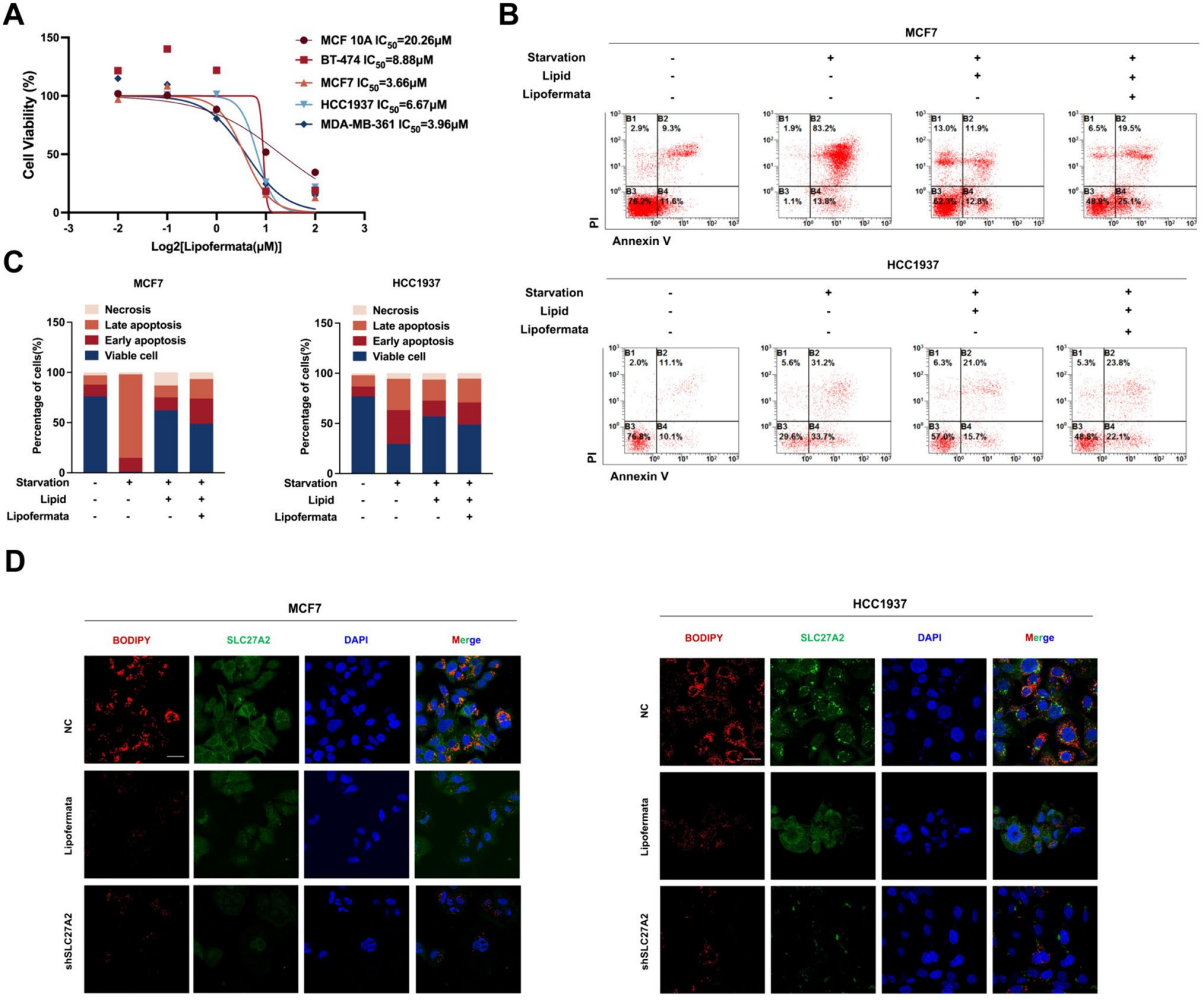
Blockade of FATP2 blockade inhibits breast cancer growth and lipid uptake. **A** The IC50 of Lipofermata in MCF 10A, MCF7, BT-474, HCC1937, and MDA-MB-361 cells was determined by CCK-8 assay and the IC50 values were calculated in GraphPad Prism 9. Data are presented as Mean ± SD (n = 3). **B**, **C** Flow cytometry was employed to assess the survival and apoptosis of MCF7 and HCC1937 cells cultured in complete media, delipidized media, and delipidized media supplemented with fatty acids. A representative experiment is shown from n = 3 independent experiments. **D** BODIPY fluorescent fatty acid uptake assay reveals reduced fluorescence uptake in MCF7 and HCC1937 cells following Lipofermata treatment and SLC27A2 knockdown. Scale bars represent 10 μm

In order to further evaluate the biological role of SLC27A2 on BC cells and its promising application as a new therapeutic target, we applied Lipofermata, a small molecule inhibitor of FATP2, in the following study. Firstly, we tested Lipofermata IC50 in SLC27A2 high-expressed BC cell lines (BT-474, MCF7, HCC1937, and MDA-MB-436) and the mammary epithelial cell line (MCF 10A). The IC50 showed that the BC cell lines were more sensitive to Lipofermata than MCF 10A, indicating that Lipofermata could be a potent BC inhibitor (Fig. 9a). We subjected cells to 6 h of starvation treatment, followed by treatment with lipid mixture or combined treatment with Lipofermata (MCF7 with 1 μM or HCC1937 with 2 μM), at which concentration showed no significant effect on cell growth and could significantly inhibit the lipid transport function, under the condition of lipid mixture supplementation. Cells were then cultured for an additional 36 h under these conditions, and apoptosis was detected by flow cytometry. We found that adding Lipofermata under the condition of lipid supplementation alone increased cell apoptosis (Fig. 9b, c). To verify whether Lipofermata could block lipid uptake, we pre-treated MCF7 and HCC1937 cell lines with serum-free culture medium for 6 h, then added 1.0 and 2.0 μM Lipofermata and extra lipid in the culture medium for 24 h, and added 1 μM BODIPY 558/568 for 2 h before fixing. Immunofluorescence showed that both Lipofermata and knockdown of SLC27A2 could inhibit long-chain FAs uptake of MCF7 and HCC1937 (Fig. 9d).

### Inhibiton of SLC27A2 surpasses tumor growth in vivo

**Fig. 10:**
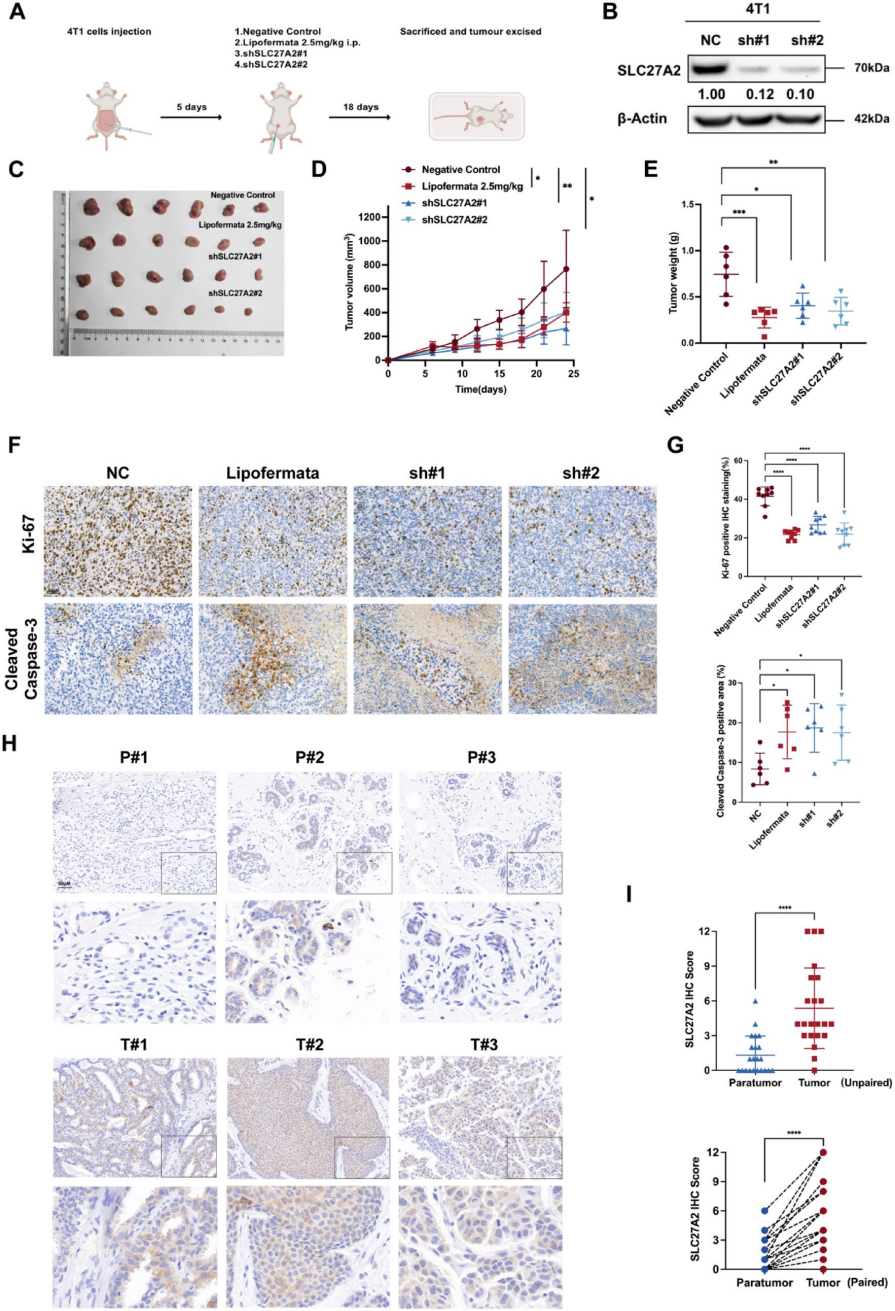
Inhibition of SLC27A2 suppresses tumor growth in vivo. **A** Schematic representation of the animal experiment protocol. **B** Tumor image of BALB/c-4T1 model captured at the end of the experiment. **C** Tracking of tumor size every 3 days in four mouse groups, depicted by the tumor growth curve and calculated tumor volume at specified time points. **D** Tumor weights post-removal. Data are presented as Mean ± SD (NC = 6, Lipofermata = 6, sh#1 = 6, sh#2 = 6); statistical analysis is conducted using one-way ANOVA with Dunnett’s multiple comparisons test; *p < 0.05, **p < 0.01, ***p < 0.001. **F** Immunohistochemical staining of tumor tissues with Ki-67 and Cleaved Caspase-3. Representative images are displayed with a scale bar of 100 μm. **G** Positive Ki-67 staining and Cleaved Caspase-3 positive area are calculated. Data are presented as Mean ± SD; statistical analysis was performed using one-way ANOVA with Dunnett’s multiple comparisons test; ****p < 0.0001. **H** Immunohistochemistry images from a tissue microarray depicting SLC27A2 expression in a panel of 24 clinically defined breast cancer tumors and para-tumor tissues. Three representative paired images are shown with a scale bar of 100 μm (*P* Paratumor, *T* Tumor). (**I**) Immunohistochemical staining for SLC27A2 in breast cancer and adjacent tissues, analyzed in unpaired and paired *t*-test comparisons; ****p < 0.0001

SLC27A2 was verified in vivo on the basis of our above findings. We injected 4T1-Negative Control, 4T1-shSLC27A2#1, and 4T1-shSLC27A2#2 cells into the mammary gland of 5-week-old BALB/c mice. In this study, mice were divided into four groups: negative control group, Lipofermata-treated group, shSLC27A2#1 group, and shSLC27A2#2 group. Lipofermata was administered every 2 days by intraperitoneal injection of 2.5 mg/kg (Fig. 10a). Knockdown of SLC27A2 in 4T1 was validated through Western blot (Fig. 10b). Both knockdown of SLC27A2 and Lipofermata treatment delayed the growth of tumors (Fig. 10c), reflected in the tumor volume (Fig. 10d) and tumor weight (Fig. 10e). Following that, we stained tumor tissues with an IHC stain for Ki-67 and Cleaved Caspase-3. Both Lipofermata-treated and shSLC27A2-treated groups indicated decreased Ki-67 expression and increased Cleaved Caspase-3 positive areas after inhibition of SLC27A2 (Fig. 10f, g). Next, we conducted IHC staining of SLC27A2 on the clinical tissue microarrays from 24 pairs of BC and adjacent non-cancerous samples. Results showed that SLC27A2 was notably higher expressed, both in paired BC tissues and unpaired tissues (Fig. 10h, i). Overall, our research provides evidence that SLC27A2 plays an important role in BC development, making it a potential target in BC therapy.

## Discussion

The absence of definitive methods for the initial diagnosis and detection of recurrence contributes significantly to the poor prognoses observed in many patients with BC. Consequently, it is critical to construct new biomarker tools for prognosis to enhance patient outcomes. Metabolic reprogramming stands as a distinctive hallmark of cancer, with nucleotide metabolism abnormalities playing a pivotal role. Directing interventions toward the metabolic disruptions observed in cancer cells has arisen as a promising approach to cancer therapy.

Nucleotide metabolism holds significant value in breast cancer therapy. For instance, targeting dUTPase inducing uracil misincorporation during chemotherapy with DNA-damaging agents can further induce tumor cell death (Davison et al. 2021). Nucleotide biosynthetic-related drugs with highly tractable features have been widely used in cancer treatment, both in monotherapy and combination therapy. Gemcitabine, an analogue of nucleoside (29,29-difluorodeoxycytidine), has exhibited both experimental and clinical efficacy in advanced or metastatic BC. Its mechanism involves halting DNA synthesis through chain termination, as well as inhibiting deoxycytidylate deaminase and ribonucleotide reductase (Cerqueira et al. 2007) (Ferrazzi and Stievano 2006). The overall impact of nucleotide metabolism on the prognosis of BC has not been reported. Our research investigated the potential prognosis biomarkers of BC, establishing a BC nucleotide metabolism signature. This signature shows promise in predicting the prognosis of BC and enhancing the effectiveness of its treatment.

Firstly, we obtained 116 differentially expressed mRNAs from TCGA-BRCA data and identified four signature genes through univariate Cox regression analysis. Afterward, by using LASSO Regression analysis, we established a prognostic model composed of four NMRGs to predict prognostic outcomes in individuals diagnosed with BC. K–M survival curves suggested that patients categorized as high-risk group experienced shorter life expectancy. The AUCs verified that this risk model possessed great predictive efficacy.

In subsequent analyses, we conducted a more comprehensive examination of high- and low-risk groups. This included tumor-related pathway analysis, gene mutation analysis, immune microenvironment landscape, and prediction of immunotherapy and chemotherapy treatment. After identifying four prognostically relevant genes and constructing a risk model, our aim was to further investigate the functions of individual genes in vitro and in vivo.

Firstly, we verified the differential expression of DCTPP1, IFNG, SLC27A2, and MYH3 in BC cells and breast epithelial cells at both the mRNA and protein levels. DCTPP1 plays a crucial role in maintaining the balance of dCTP and the metabolism of deoxycytidine analogues by hydrolyzing dCTP into dCMP and pyrophosphate (Requena et al. 2014)(Martinez-Arribas et al. 2020). Prior studies have indicated that DCTPP1 is upregulated in specific cancer types and correlates with enhanced proliferation and invasion capabilities of tumor cells. For example, higher expression of DCTPP1 is closely related to the worse prognosis of patients with BC and prostate cancer (Brown et al. 2017) (Song et al. 2015)(Lu et al. 2018). Interferon gamma (IFN-γ) released by CD8^+^ T cells downregulates the expression of two subunits, SLC3A2 and SLC7A11, of the glutamate-cysteine antiporter system, impairing tumor cell uptake of cysteine, thereby promoting lipid peroxidation and ferroptosis in tumor cells (Lang et al. 2019). MYH3 is a type of myosin, which can convert chemical energy into mechanical energy through the hydrolysis of ATP. It is widely distributed in eukaryotic cells, and its relationship with tumor occurrence and development remains to be further explored (Toydemir et al. 2006).

Given the notable upregulation of SLC27A2 at both RNA and protein levels in BC cell lines, and the absence of reported biological functions and roles in BC, we selected SLC27A2 for additional investigation. Through both in vitro and in vivo methods, we observed that the reduction of SLC27A2 suppressed proliferation, increased cell apoptosis, and also affected the transport of long-chain FAs, as well as the expression of key molecules involved in nucleotide metabolism. This suggests that SLC27A2 may have tumor-promoting properties.

SLC27A2 encodes FA transport protein known as FATP2, alternatively named very long-chain acyl-coenzyme A (CoA) synthetase 1 (ACSVL1) (Uchiyama et al. 1996). It has two main functions: one as a FA transporter, and the other as a long-chain acyl-CoA synthetase. Therefore, SLC27A2 plays a crucial role in controlling the uptake of external FAs and maintaining intracellular lipid homeostasis (Ahowesso et al. 2015). Nucleotide and lipid metabolism are tightly regulated processes that respond to cellular energy status, metabolic demands, and signaling pathways. Aberrant lipid metabolism, such as increased FA synthesis or altered lipid composition, can impact nucleotide synthesis and signaling pathways involved in cellular proliferation and survival.

Research has found that carnitine palmitoyltransferase 1 isoform (CPT1A), an important rate-limiting step of FA oxidation, which shuttles long-chain FAs into mitochondria, could impact the utilization of fatty acid-derived carbons in de novo nucleotide synthesis essential for DNA replication (Schoors et al. 2015). CPT1A affects tumor cell proliferation by influencing cellular and nucleotide metabolism (Tang et al. 2022). The role of SLC27A2 in BC and its relationship between nucleotide metabolism and lipid metabolism have not been reported. FATPs (FATP1–6, encoded by SLC27A1-6) could help import FAs, and more research indicates the crucial role of FA transporters in oncogenesis and chemoresistance (Kazantzis and Stahl 2012).

FATP2 in melanoma cells uptakes lipids from senescent fibroblasts to help resist targeted therapy. Blocking FATP2 in melanoma cells can inhibit lipid accumulation and disrupt mitochondrial metabolism (Alicea et al. 2020). MYCN upregulates SLC27A2 expression to enhance FAs uptake, supporting neuroblastoma survival and tumor growth. Feng et al. reported that the overexpression of SLC27A2 was observed in differentiated thyroid carcinoma and affects cell proliferation and differentiation (Feng et al. 2022).

SLC27A2 exhibits a critical function not only in BC cells but also in shaping the tumor microenvironment. Studies have demonstrated that inhibiting the upregulation of SLC27A2 in polymorphonuclear myeloid-derived suppressor cells can weaken their immunosuppressive effects and inhibit tumor progression (Veglia et al. 2019). As a novel target for cancer research and treatment, further exploration of SLC27A2 expression in immune cells within the tumor immune microenvironment is warranted. Consequently, SLC27A2 emerges as an outstanding therapeutic target for regulating tumor growth across various malignant cell types.

While scientists have elucidated the biological functions of lipid transporters like SLC27A2, their potential as significant drug targets remains largely unexplored. Ahowesso et al. identified a specific FA transport inhibitor, CB16.2, also known as Lipofermata, through high-throughput screening methods, effectively inhibiting the uptake of long-chain FAs in vivo (Ahowesso et al. 2015). Black et al. applied a naturally occurring splice variant of FATP2, FATP2b, to further validate that Lipofermata can effectively attenuate or block the transport of FAs without inhibiting intrinsic acyl CoA synthetase activity (Black et al. 2016). Treatment with the FATP2 inhibitor Lipofermata in hepatitis B virus X (HBx) expressed cell lines and mice improved HBx-triggered lipid accumulation and alleviated oxidative stress and inflammation associated with lipid accumulation (Lu et al. 2023). However, the application of Lipofermata in tumors remains unexplored. Therefore, we applied Lipofermata in a mouse BC model for the first time and found that Lipofermata not only inhibited lipid uptake in BC cells in vitro but also exerted antitumor effects in vivo.

Although our research has identified a correlation between nucleotide metabolism-related molecules and the prognosis of BC for the first time, there are still some limitations to this study. Due to the limited sample scale, we did not analyze the prognostic ability of tumors based on subtypes. Through this study, we have embarked on preliminary explorations into the biological functions of FATP2 in BC, and we are poised to delve deeper into elucidating the molecular mechanisms in our future investigations.

In summary, our study selected four NMRGs (DCTPP1, IFNG, SLC27A2, and MYH3) and constructed a prognostic risk model for BC. The results indicate that all four genes are independent prognostic factors, and the prognostic model built with these genes can accurately predict the prognosis of BC. Additionally, the prognostic model can accurately predict the extent of immune infiltration and responsiveness to drugs in patients. Among these genes, SLC27A2 was selected as a target for biological experimental validation, and both in vitro and in vivo experiments confirmed the significant role of SLC27A2 in the development of tumors. Overall, our findings are of great significance for studying the molecular pathways and mechanisms of BC, as well as for the development of treatments and prognostic assessment for BC.

### Supplementary Information

Below is the link to the electronic supplementary material.

Supplementary file1 (PDF 755 KB)

Supplementary file2 (DOCX 18 KB)

## Data Availability

All data and R scripts for this research can be obtained from the corresponding authors. In this study, the data were obtained from two publicly available databases: The Cancer Genome Atlas (https://portal.gdc.cancer.gov/), the GEO dataset (http://www.ncbi.nlm.nih.gov/geo/) and the Comparative Toxicogenomics Database (http://ctdbase.org/).
