# Supplementary material for: Risk assessment model based on nucleotide metabolism-related genes highlights SLC27A2 as a potential therapeutic target in breast cancer
Source: J Cancer Res Clin Oncol. 2024 May 16;150(5):258. doi: 10.1007/s00432-024-05754-x (PMC11098904; doi:10.1007/s00432-024-05754-x)
Supplement: Supplementary file 1 — Supplementary file1 (PDF 755 KB) [file 432_2024_5754_MOESM1_ESM.pdf]

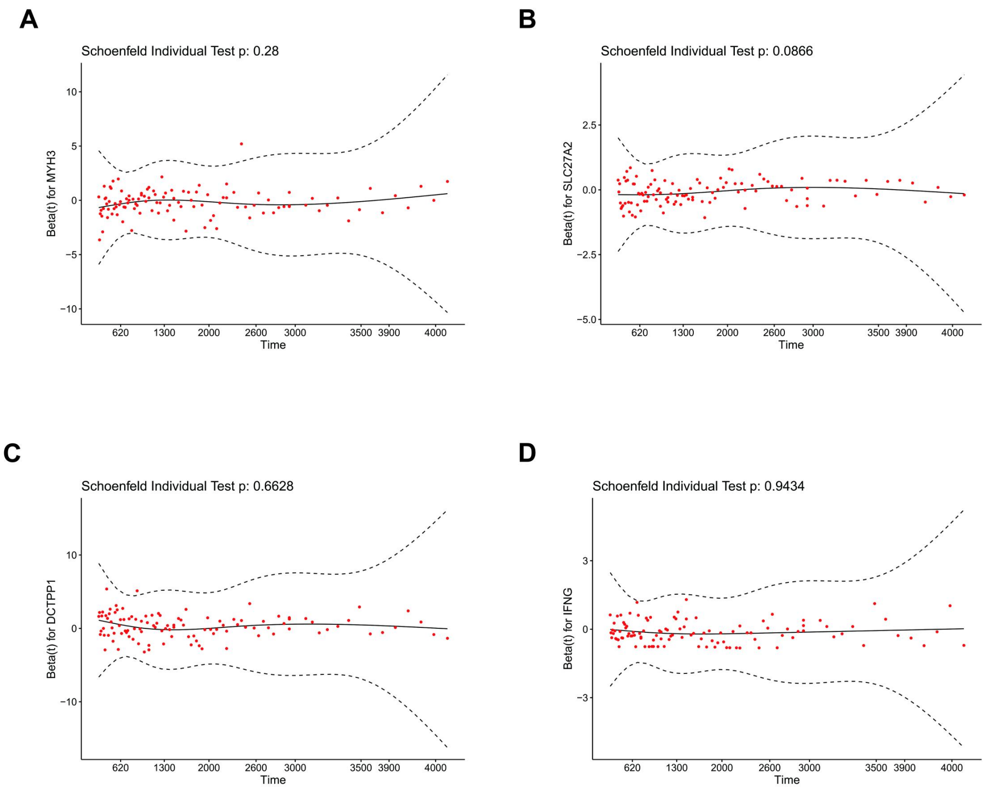

Figure S1. Schoenfeld residuals tests of four signature genes obtained from univariate Cox regression analysis. Schoenfeld residuals tests of MYH3 (A), SLC27A2 (B), DCTPP1 (C) and IFNG (D).

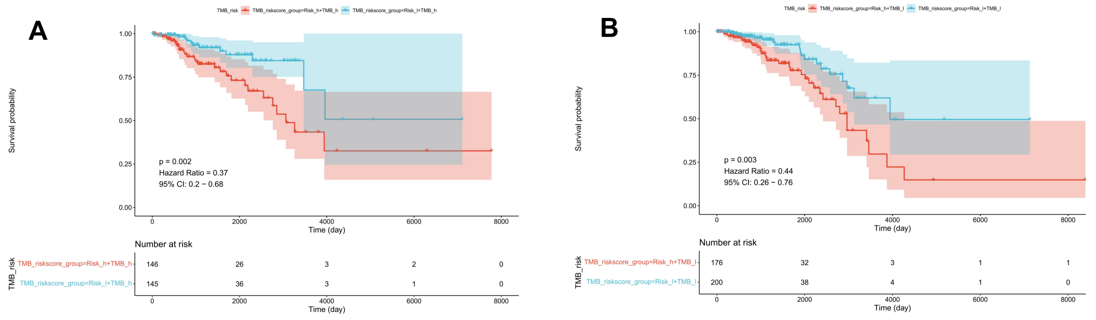

Figure S2. K-M analysis of low- and high-risk groups in low- and high- TMB groups. (A) Survival analysis of overall survival (OS) between the Risk_high+TMB_high and Risk_low+TMB_high group. (B) Survival analysis of OS between the Risk_high+TMB_low and Risk_low+TMB_low group.
