# Supplementary material for: Risk assessment model based on nucleotide metabolism-related genes highlights SLC27A2 as a potential therapeutic target in breast cancer
Source: J Cancer Res Clin Oncol. 2024 May 16;150(5):258. doi: 10.1007/s00432-024-05754-x (PMC11098904; doi:10.1007/s00432-024-05754-x)
Supplement: Supplementary file 2 — Supplementary file2 (DOCX 18 KB) [file 432_2024_5754_MOESM2_ESM.docx]

Supplementary Table S1

| Reagents | Source | Identifier |
| --- | --- | --- |
| Anti-mouseSLC27A2 | Santa Cruz | Cat#sc-393906 |
| Anti-rabbit MYH3 | Proteintech | Cat#22287-1-AP |
| Anti-rabbit IFN Gamma | Proteintech | Cat#15365-1-AP |
| Anti-rabbit DCTPP1 | Proteintech | Cat#16684-1-AP |
| Anti-rabbit p-Rb(Ser807/811) | CST | Cat#8516 |
| Anti-rabbit p-Rb(Ser780) | CST | Cat#8180 |
| Anti-mouse Rb | CST | Cat#9309 |
| Anti-mouse Cyclin D1 | Proteintech | Cat#60186-1-Ig |
| Anti-Rabbit c-Myc | CST | Cat#18583 |
| Anti-rabbit Bax | Proteintech | Cat#50599-2-IG |
| Anti-rabbit Bcl-2 | CST | Cat#3498 |
| Anti-rabbit GAPDH | CST | Cat#5174 |
| Anti-mouse β-Actin | Boster | Cat#BM0627 |

Antibodies used for Western Blot

Supplementary Table S2

Primer sequences

| Gene name | Species | Primer sequence (5‘-3’) |
| --- | --- | --- |
|  |  |  |
| F-DCTPP1 | Human | CGCCTCCATGCTGAGTTTG |
| R-DCTPP1 | Human | CCAGGTTCCCCATCGGTTTTC |
| F-SLC27A2 | Human | TACTCTTGCCTTGCGGACTAA |
| R-SLC27A2 | Human | CCGAAGCAGTTCACCGATATAC |
| F-MYH3 | Human | GTTAAGGGCCTGAGGAAGTATG |
| R-MYH3 | Human | TTATCCACCAGATCCTGCAATC |
| F-IFNG | Human | TCGGTAACTGACTTGAATGTCCA |
| R-IFNG | Human | TCGCTTCCCTGTTTTAGCTGC |
| F-β-Actin | Human | CATGTACGTTGCTATCCAGGC |
| R-β-Actin | Human | CTCCTTAATGTCACGCACGAT |
| F-NT5E | Human | CCAGTACCAGGGCACTATCTG |
| R-NT5E | Human | TGGCTCGATCAGTCCTTCCA |
| F-ENTPD1 | Human | AGGTGCCTATGGCTGGATTAC |
| R-ENTPD1 | Human | CCAAAGCTCCAAAGGTTTCCT |
| F-ADSS2 | Human | TGGTGCCTTTCCTACAGAGC |
| R-ADSS2 | Human | TGAGCAAAACGAGGTCCAACC |
| F-ADA | Human | GCCTTCGACAAGCCCAAAGTA |
| R-ADA | Human | CTCTGCTGTGTTAGCTGGGAG |
| F-DPYD | Human | TCAAGCACACGACTCTTGGTG |
| R-DPYD | Human | CATACCATTCCACAAGTCAGACC |
| F-CAD | Human | CCATGCACTAGACAGCCAAGA |
| R-CAD | Human | CGGCTCAGTGTGGATACGAC |
| F-GART | Human | GTGTCTGTGTTTCACTGATGGC |
| R-GART | Human | GCTGGACAATAGGCTCCCATT |
| F-PRPS1 | Human | ATCTTCTCCGGTCCTGCTATT |
| R-PRPS1 | Human | TGGTGACTACTACTGCCTCAAA |
| F-IMPDH2 | Human | AGTGGCTCCATCTGCATTAC |
| R-IMPDH2 | Human | AAAGCGCCGTGCATACT |
